# P-Selectin Inhibition and the Structure–Activity Relationship of Sea Cucumber-Derived Fucosylated Glycosaminoglycan Oligosaccharides

**DOI:** 10.3390/md24050177

**Published:** 2026-05-14

**Authors:** Sujuan Li, Lisha Lin, Lian Yang, Ying Pan, Na Gao, Ronghua Yin, Chunyu Zeng, Jinhua Zhao

**Affiliations:** 1 Department of Cardiology, Army Medical Center, Army Medical University, Chongqing 400042, China; lisujuan1988@126.com; 2 Key Laboratory of Geriatric Cardiovascular and Cerebrovascular Disease Research, Ministry of Education of China, Chongqing 400042, China; 3 State Key Laboratory of Phytochemistry and Natural Medicines, Kunming Institute of Botany, Chinese Academy of Sciences, Kunming 650201, China; linlisha@mail.kib.ac.cn (L.L.); yanglian@mail.kib.ac.cn (L.Y.); 4 School of Pharmaceutical Sciences, South-Central Minzu University, Wuhan 430074, China; 13140364802@163.com (Y.P.); zhaojhscu@163.com (J.Z.)

**Keywords:** fucosylated glycosaminoglycan, oligosaccharide, P-selectin inhibitor, structure-activity relationship, inflammatory diseases

## Abstract

The selectin family constitutes a well-known class of immune-regulatory molecules, among which P-selectin has emerged as a therapeutic target for inflammatory thrombotic diseases due to its capacity to mediate the adhesion between multiple immune cell subsets and endothelial cells. Currently, small-molecule or glycomimetic inhibitors targeting P-selectin have stalled in Phase III clinical trials, with a common limitation being their weak binding affinity to P-selectin. In this study, in vitro competitive binding assays were employed to evaluate the inhibitory effects of structurally distinct fucosylated glycosaminoglycan (FG) oligosaccharides, derived from sea cucumbers, on the interaction between P-selectin and its ligands. A potent inhibitor, the nonasaccharide Ta-9-2 (featuring a novel disaccharide side chain), was identified. Biolayer interferometry (BLI) analysis further confirmed its high binding affinity to P-selectin, with a *K*_D_ of 83.92 nM. Structure–activity relationship (SAR) analysis reveals that the appropriate glycan chain length, the novel disaccharide side chain (Gal_4S6S_-α1,2-L-Fuc_3S_-α1,3), and the favorable sulfation pattern (Fuc_2S4S_) serve as the molecular basis for potent P-selectin inhibition. This study provides a robust theoretical foundation for the structural optimization of glycomimetic targeting P-selectin, while also offering a new opportunity for the development of high-efficacy drug candidates.

## 1. Introduction

The selectin family is a well-known class of immunomodulatory molecules characterized as calcium-dependent type I transmembrane glycoproteins. This family comprises three members, categorized by their primary expression sites: E-selectin (expressed on endothelial cells), P-selectin (predominantly expressed on platelets and endothelial cells), and L-selectin (predominantly expressed on leukocytes) [1]. Notably, P-selectin is stored in Weibel–Palade bodies of endothelial cells and α-granules of platelets, and undergoes rapid translocation to the plasma membrane surface upon cellular activation [2,3,4]. Leukocyte homeostasis and recruitment to inflammatory sites are governed by multiple interactions among the three selectins, with P-selectin exerting a dominant functional role [5]. The binding of P-selectin to its primary physiologically ligand P-selectin glycol-protein ligand-1 (PSGL-1), which is constitutively expressed on leukocytes, mediates the rolling and adhesion of leukocytes to endothelial cells, which is a critical step in the development and progression of inflammation, metastasis, and thrombosis [6,7]. PSGL-1 is a sialic acid-rich transmembrane adhesion molecule, harboring approximately 70 extracellular serine (Ser) and threonine (Thr) residues that serve as potential O-glycosylation sites, in addition to three conserved asparagine (Asn) residues corresponding to potential N-glycosylation sites. Structural and functional studies on the molecular basis of P-selectin–human PSGL-1 interaction have demonstrated that tyrosine sulfation of the PSGL-1 N-terminal domain, along with fucosylation and sialyl Lewis X (sLe^X^) epitopes on specific O-glycans, make the most significant contributions to their high-affinity binding [8,9,10].

To date, inhibitors targeting the P-selectin/PSGL-1 pathway have been categorized by their structural features into the following major classes: anti-P-selectin antibodies, anti-PSGL-1 antibodies, recombinant PSGL-1-immunoglobulin fusion proteins, anti-P-selectin aptamers, glycomimetics, natural glycans and their derivatives, glycopeptides, glycolipids, and small molecules. Among these, only the P-selectin-blocking monoclonal antibody Crizanlizumab (SelG1; Adakveo [Novartis]) has received US Food and Drug Administration approval (in 2019) for the prevention of sickle cell vaso-occlusive crises; all others were discontinued in Phase III clinical trials due to safety concerns or suboptimal efficacy [11,12]. A common limitation of these inhibitors is their weak binding affinity to P-selectin. Thus, enhancing binding affinity represents a primary objective in the structural optimization of P-selectin inhibitors [13].

Fucosylated glycosaminoglycan (FG), also known as fucosylated chondroitin sulfate (FCS), is a glycosaminoglycan with a distinct structure derived from sea cucumber, which consists of a chondroitin sulfate-like backbone and unique branches of sulfated fucoses. Accumulating evidence demonstrates that FG exhibits wide-ranging bioactivities, including anticoagulant, antithrombotic, antiviral, antitumor, and antiplatelet effects [14,15,16,17]. While natural FG possesses potent bioactivity, it is associated with side effects such as platelet aggregation induction and poor pharmacological target selectivity. Notably, depolymerized FG and its derived oligosaccharides, obtained via controlled depolymerization, eliminate these adverse effects, retain core pharmacological activities, and enhance target selectivity. Specifically, the depolymerized FG with a molecular weight (Mw) of ~5.3 kDa (LFG-53/YB209) exerts anticoagulant and antithrombotic effects by potently inhibiting the intrinsic factor tenase, with a lower bleeding risk compared to classic anticoagulants (e.g., enoxaparin) [18,19]. It has been granted an Investigational New Drug (IND) approval (IND 153953) by the U.S. Food and Drug Administration (FDA) for clinical trials. FG has also been reported to inhibit P-selectin [20,21]. Owing to the structural diversity of FG-depolymerized oligosaccharides derived from distinct sea cucumber species and the challenges associated with their preparation, a comprehensive investigation into their structure–activity (SAR) relationship is highly warranted but remains unexplored.

In the present study, in vitro competitive binding assays were employed to evaluate the inhibitory effects of a series of FG oligosaccharides on P-selectin binding to its ligands. A potent inhibitor (nona-saccharide Ta-9-2), featuring a novel disaccharide side chain, was identified and its binding affinity (*K*_D_ = 83.92 nM) was determined using biolayer interferometry (BLI). SAR analysis revealed that the optimal chain length, the novel disaccharide side chain (Gal_4S6S_-α1,2-L-Fuc_3S_-α1,3), and the favorable sulfation pattern (Fuc_2S4S_) constitute the structural basis for high-affinity P-selectin recognition. This study presents, for the first time, a comprehensive SAR analysis of FG oligosaccharides inhibiting P-selectin. It also indicates the promising potential of FG oligosaccharides as a source for developing high-efficacy anti-P-selectin drugs and provides a new opportunity for the treatment of thromboinflammation diseases.

## 2. Results

### 2.1. Structural Characteristics of FG Oligosaccharides

**Figure 1:**
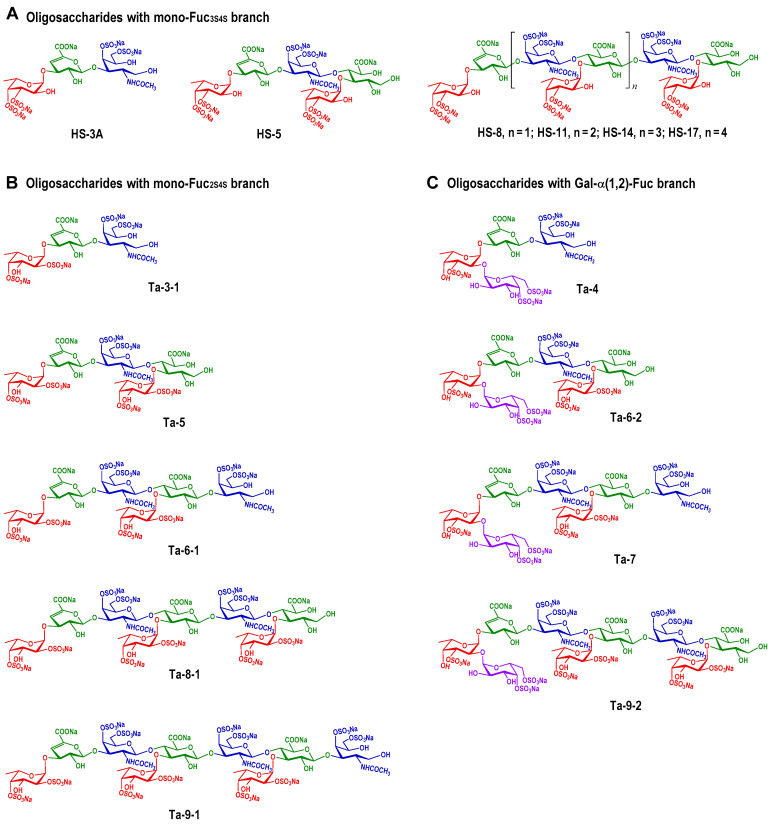
Structures of oligosaccharides purified from the depolymerized products of FG derived from *H. fuscopunctata* (**A**) and *T. ananas* (**B**,**C**).

According to our previous study, native FG had a backbone consisting of repeated {4-D-glucuronic acid (GlcA)-β 1,3-N-acetyl-D-galactosamine (GalNAc)-β 1,} disaccharide units, and abundant sulfated α-L-fucose (FucS) branches linked to the C3 of each GlcA residue. Oligosaccharides with branches of 3,4-di-O-sulfated fucose (Fuc_3S4S_), including tri- (HS3A), penta- (HS-5), octa- (HS-8), undeca-(HS-11), tetradeca-(HS-14), and heptadeca (HS-17)-saccharides, were purified from the depolymerized products of FG derived from *Holothuria fuscopunctata* (*H. fuscopunctata*), with structures shown in Figure 1A [19]. From the depolymerized products of FG derived from *Thelenota ananas* (*T. ananas*), oligosaccharides with branches of 2,4-di-O-sulfated fucose (Fuc_2S4S_), including tri- (Ta-3-1), penta- (Ta-5), hexa- (Ta-6-1), octa- (Ta-8), and nona- (Ta-9-1) saccharides, or those with a novel disaccharide-branch composed of galactose (Gal) and Fuc (D-Gal_4S6S_-α1, 2-L-Fuc_3S_-α1, 3-), including tetra- (Ta-4), hexa- (Ta-6-2), hepta- (Ta-7), and nona- (Ta-9-2) saccharides, were purified, as shown in Figure 1B [22]. Sialyl Lewis X (sLe^X^) is a tetrasaccharide composed of sialic acid (N-Acetylneuraminic acid, Neu5Ac), Gal, N-acetylglucosamine (GlcNAc), and Fuc arranged in a specific sequence [23]. Fondaparinux (Fpx) is the α-linked methyl glycoside of the heparin pentasaccharide fragment, which consists of GlcNAc, GlcA, and iduronicacid (IdoA) [24]. The molecular weights of the oligosaccharides are presented in Table 1.

### 2.2. FG Oligosaccharides Inhibit the Binding of P-Selectin to sLe^X^

**Figure 2:**
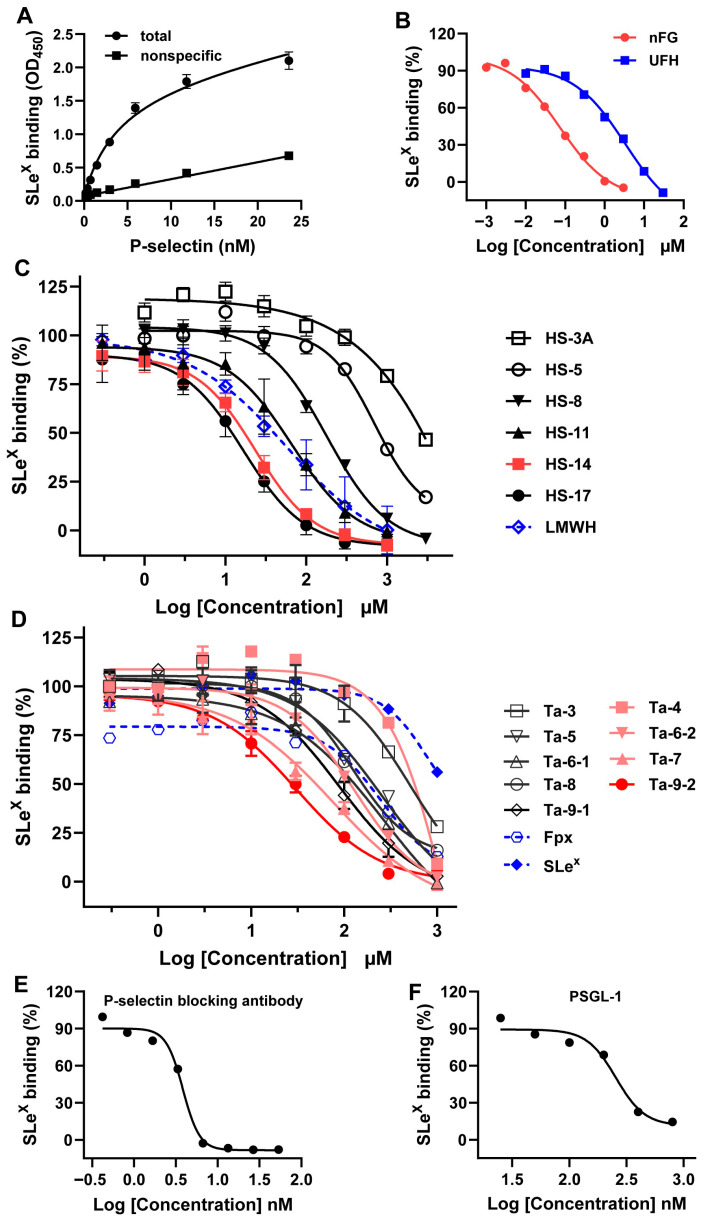
FG oligosaccharides inhibit the binding of P-selectin to sLe^X^. (**A**) Binding-saturation curve of sLe^X^ to P-selectin. A series of concentrations of P-selectin bound to sLe^X^-PAA-B immobilized on a streptavidin plate. *K*_D_ = 4.011 nM (95% CI, 2.752–5.270). *n* = 4. (**B**–**F**) Competition binding assays were performed by detecting the binding of sLe^X^-PAA and P-selectin (1 μg/mL) in the presence of a series of concentrations of inhibitors. The relative binding response was calculated by normalizing each signal to the control (without inhibitors). IC_50_ and *K*_i_ (inhibition dissociation constant) were determined by fitting the log (inhibitor) vs. response curves equation (a 4-parameter dose response curve) and the one site-fit *K*_i_ equation using the GraphPad Prism 10.0 software, respectively.

sLe^X^ is a canonical carbohydrate ligand for selectins and represents a functionally essential glycan epitope on PSGL-1 that directly mediates high-affinity binding to P-selectin [23]. The competitive inhibitory effect of FG oligosaccharides on P-selectin binding to sLe^X^-PAA was evaluated via microplate binding assays, and half-maximal inhibitory concentrations (IC_50_) were calculated. To determine the inhibition dissociation constants (*K*_i_) for each oligosaccharide, the binding saturation curve of P-selectin to sLe^X^-PAA was first measured, yielding an equilibrium dissociation constant (*K*_D_) of 4.011 nM (95% confidence interval [CI], 2.752–5.270) (Figure 2A). Subsequent compound inhibition assays were performed to compute *K*_i_ values for each test compound and control; detailed calculation methods are described in the Section 4.

The native FG exhibited potent inhibition of P-selectin binding to sLe^X^-PAA, with an IC_50_ of 0.07409 μM (95% CI, 0.03579–0.1534), representing a 44.86-fold increase in potency relative to heparin (IC_50_ = 3.324 μM; 95% CI, 1.091–10.13) (Figure 2B). These results indicate that nFG contains structurally conserved motifs conferring high-affinity, competitive inhibition of P-selectin.

To identify structurally optimized P-selectin inhibitors, FG-derived oligosaccharides with defined degrees of polymerization and side-chain architectures were generated through β-eliminative cleavage and chromatographic purification, followed by quantitative assessment of their inhibitory activity against P-selectin–sLe^X^ binding. A summary of the SAR analysis is presented in Figure 2C,D and Table 1.

Oligosaccharides bearing Fuc_3S4S_ monosaccharide side chains (HS-3A, HS-5, HS-8, HS-11, HS-14, HS-17; structures in Figure 1A), Fuc_2S4S_ monosaccharide side chains (Ta-3-1, Ta-5, Ta-6-1, Ta-8, Ta-9-1; structures in Figure 1B), and complex disaccharide side chains (Ta-4, Ta-6-2, Ta-7, Ta-9-2; structures in Figure 1C) all demonstrated a clear polymerization-dependent enhancement in inhibitory potency, with IC_50_ values decreasing from 701.1 to 16.62 μM, 514.8 to 89.02 μM, and 128.1 to 30.26 μM, respectively. HS-17 and HS-14 exhibited statistically indistinguishable activity (*p* > 0.05), both surpassing that of low-molecular-weight heparin (LMWH) matched for molecular weight. In contrast, very short oligosaccharides, including trisaccharides and tetrasaccharides (HS-3A, Ta-3-1, Ta-4, and sLe^X^)—showed negligible inhibition, with IC_50_ values exceeding 500 μM. Oligosaccharides featuring the novel disaccharide side chain consistently displayed superior potency relative to their monosaccharide-side-chain counterparts. Notably, Ta-9-2 (IC_50_ = 30.26 μM; 95% CI, 22.42–40.86) was significantly more potent than Ta-8 (IC_50_ = 125.5 μM; 95% CI, 101.4–155.5) and Ta-9-1 (IC_50_ = 89.02 μM; 95% CI, 58.88–134.6), and its activity was comparable to that of higher-molecular-weight LMWH (IC_50_ = 40.23 μM; 95% CI, 21.41–152.8). Furthermore, although sulfate regiochemistry had no discernible effect on inhibitory activity in trisaccharide analogs (HS-5 vs. Ta-5), it conferred a marked advantage in octasaccharides: Ta-8 (Fuc_2S4S_) exhibited significantly stronger inhibition than HS-8 (Fuc_3S4S_), with IC_50_ values of 125.5 μM (95% CI, 101.4–155.5) and 179.1 μM (95% CI, 149.2–215.1), respectively. Collectively, these findings demonstrate that the high-affinity inhibition of P-selectin–sLe^X^ binding by FG oligosaccharides is governed by three interdependent structural determinants: (i) sufficient glycan chain length, (ii) the presence of a disaccharide side chain (Gal_4S6S_-α1,2-L-Fuc_3S_-α1,3), and (iii) sulfate positioning—particularly when chain length permits optimal spatial presentation of sulfated epitopes.

The anti-P-selectin monoclonal antibody and PSGL-1 were also evaluated, with IC_50_ values of 3.841 nM (95% CI 3.105–4.752) and 262.2 nM (95% CI 106.7–609.5), respectively (Figure 2E,F and Table 1). *K*_i_ values are provided in Table 1 to enable precise cross-comparison with inhibitors developed by other groups.

### 2.3. FG Oligosaccharides Inhibit the Binding of P-Selectin to HL-60 Cells

**Figure 3:**
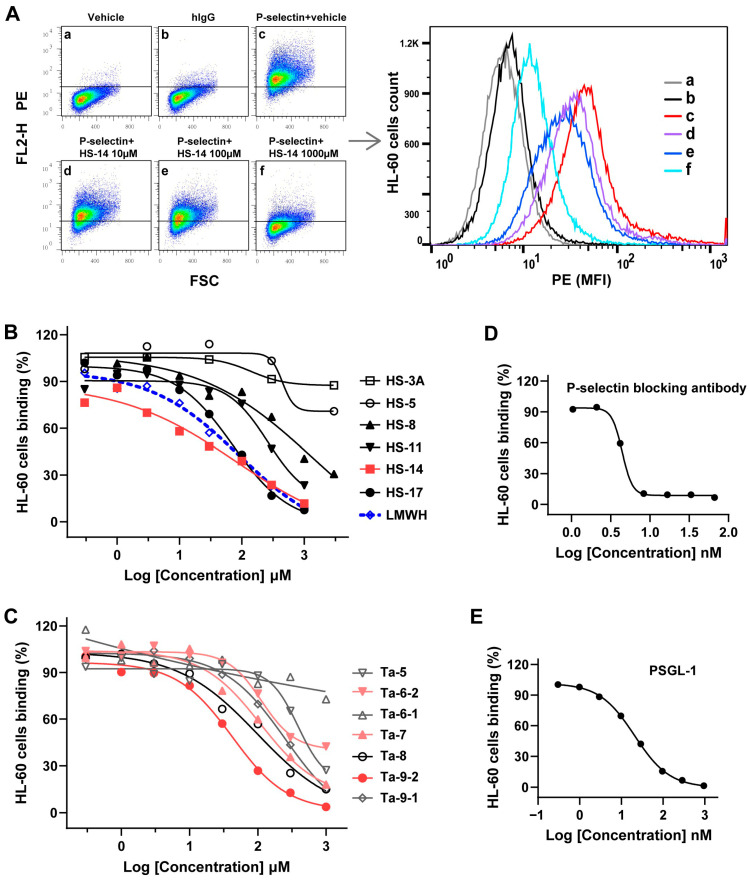
FG oligosaccharides inhibit the binding of P-selectin to HL-60 cells. Competition binding assays were performed by detecting the binding of HL-60 and P-selectin (1.25 μg/mL) in the presence of a series of concentrations of inhibitors. (**A**) Flow cytometry analysis strategy exemplified by oligosaccharide HS-14. (**B**–**E**) The relative binding response was calculated by normalizing each signal to the control (without inhibitors). IC_50_ were determined by fitting the log (inhibitor) vs. response curves equation (a 4-parameter dose response curve).

Under a more physiologically relevant screening paradigm, the competitive inhibitory effects of structurally distinct FG oligosaccharides on P-selectin binding to HL-60 cells were evaluated, with results summarized in Figure 3 and Table 1. Macroscopically, each series of FG oligosaccharides exhibited a molecular weight-dependent enhancement in inhibitory activity, consistent with their effects on P-selectin/sLe^X^ binding. Notably, the nonasaccharide Ta-9-2 (Mw = 2724), featuring a novel side chain, displayed unexpectedly potent inhibition, with an IC_50_ of 41.24 μM (95% CI, 33.73–50.42)—approximately 2-fold more active than the higher-molecular-weight HS-14 (Mw = 4374, IC_50_ = 78.77 μM; 95% CI, 38.92–113.6), HS-17 (Mw = 5329, IC_50_ = 71.03 μM; 95% CI, 41.22–122.4), and LMWH (Mw ~4500, IC_50_ = 90.20 μM; 95% CI, 53.10–165.9).

Additionally, Ta-8 (Fuc_2S4S_) showed a ~10-fold greater inhibitory activity than HS-8 (Fuc_3S4S_), with IC_50_ values of 97.54 μM (95% CI, 51.42–216.4) and >1000 μM, respectively. Its activity was comparable to that of the higher-molecular-weight HS-14, HS-17, and LMWH, indicating that the Fuc_2S4S_ sulfation pattern confers a functional advantage in competitive inhibition. However, for shorter-chain oligosaccharides (e.g., Ta-5, Ta-6-1, Ta-6-2)—even those with novel side chains or favorable sulfation profiles—inhibitory activity was negligible (IC_50_ > 1000 μM) and incomplete, suggesting that novel disaccharide side chains (Gal_4S6S_-α1,2-L-Fuc_3S_-α1,3) and sulfation patterns of monosaccharide side chains do not enhance activity in the absence of sufficient chain length. Thus, optimal chain length is a prerequisite for functional activity. FG oligosaccharides compete with endogenous macromolecular ligands for binding to P-selectin, requiring a spatial conformation coordinated by glycan chain length, side-chain architecture, and sulfation pattern.

### 2.4. Binding Kinetic Characteristics of FG Oligosaccharides with P-Selectin

Biolayer interferometry (BLI) was employed to real-time monitor the association-dissociation kinetics of FG oligosaccharides binding to P-selectin, enabling characterization of their binding dynamics and further dissection of how chain length, side-chain architecture, and sulfation pattern regulate P-selectin binding.

**Figure 4:**
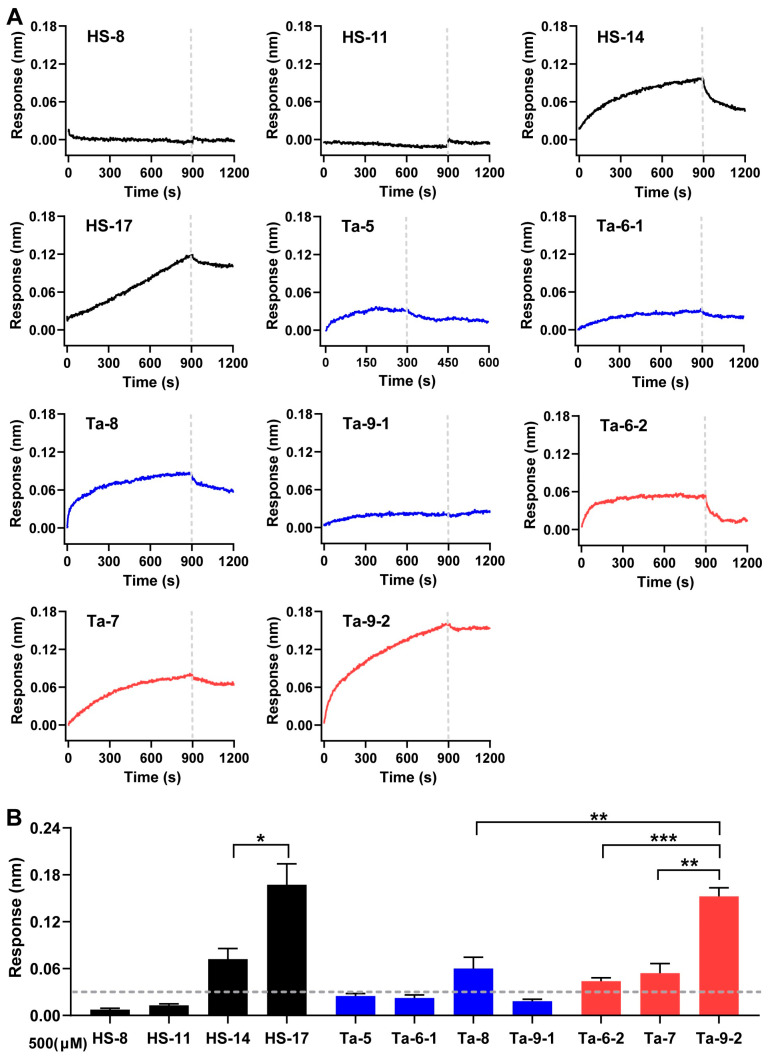
Binding response of FG oligosaccharides to P-selectin. (**A**) A single concentration (500 µM) of FG oligosaccharides was incubated with P-selectin immobilized on Protein A biosensors. Response curves are shown after subtraction of the background control. (**B**) Binding responses were calculated. *n* = 3; error bars represent SD; * *p* < 0.05,** *p* < 0.01, or *** *p* < 0.001.

First, the binding signals of various FG oligosaccharides (500 μM) to P-selectin were measured (Figure 4A,B). The nonasaccharide Ta-9-2, featuring a novel disaccharide side chain (Gal_4S6S_-α1,2-L-Fuc_3S_-α1,3), exhibited a prominent binding signal—significantly higher than that of shorter-chain analogs with identical side chains (Ta-7, Ta-6-2), and monosaccharide side-chain counterparts (Ta-8 [Fuc_2S4S_]), indicating the important role of chain length and the disaccharide side chain. Notably, although oligosaccharides (HS-11, HS-14, and HS-17 [Fuc_3S4S_]) have a longer chain length, they showed weaker binding activity than Ta-9-2, further proving that the disaccharide side chain in Ta-9-2 is essential for its activity. Compared with HS-8 (Fuc_3S4S_), Ta-8 (Fuc_2S4S_) showed much stronger binding, indicating the favorable sulfation pattern of Fuc_2S4S_. Additionally, HS-17 produced a binding signal but failed to reach saturation within the same timeframe as other oligosaccharides, displaying a linear binding profile, which indicates that the signal of HS-17 arose primarily from non-specific interactions driven by its excessively long glycan chain rather than specific binding with P-selectin. In P-selectin/sLe^X^ or HL-60 cell binding assays, the inhibitory activity observed for HS-17 and other oligosaccharides lacking detectable BLI binding signals are likely due to steric hindrance effects arising from their glycan chains.

**Figure 5:**
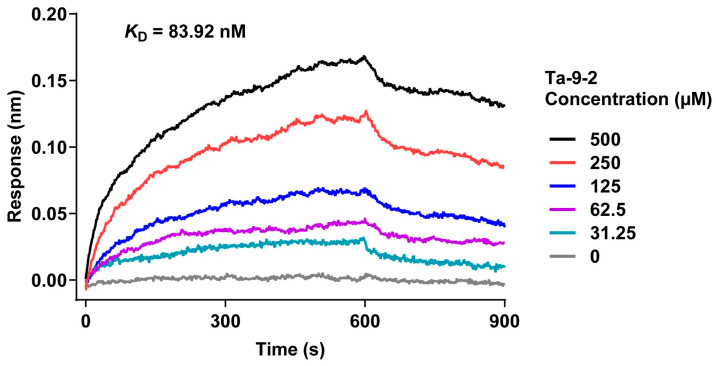
Binding kinetics of the FG oligosaccharide Ta-9-2 to P-selectin. Gradient concentrations of Ta-9-2 were incubated with P-selectin immobilized on Protein A biosensors. The binding curves were globally fitted to a 1:1 binding model using the Octet Analysis Studio 13 software. *K*_D_ = 83.92 ± 1.084 nM (*K*_D_ is the mean ± SEM).

Additionally, the binding signals of P-selectin to Ta-9-2 at gradient concentrations (31.25–500 μM) were further studied (Figure 5). Kinetic parameters were determined via global fitting of the binding curves using a 1:1 binding model. The results revealed that Ta-9-2 could bind to P-selectin with a high affinity, *K*_D_ of 83.92 ± 1.084 nM, an association rate constant (*k*_on_) of 1.563 × 10^4^ M^−1^s^−1^, and a dissociation rate constant (*k*_off_) of 1.311 × 10^−3^ s^−1^, indicating approximately 4-fold higher affinity than that of the physiological ligand PSGL-1 (*K*_D_ = 320 nM, *k*_on_ = 4.4 × 10^6^ M^−1^s^−1^, *k*_off_ = 1.4 s^−1^ [25]). Notably, Ta-9-2 displays a slow dissociation rate, whereas PSGL-1 undergoes a rapid association–dissociation cycle with P-selectin.

Taken together, these results confirm that the P-selectin binding capacity of FG oligosaccharide requires specific structural features, including the appropriate glycan chain length, a favorable disaccharide side chain (Gal_4S6S_-α1,2-L-Fuc_3S_-α1,3), and sulfation pattern (Fuc_2S4S_).

## 3. Discussion

Blockade of the interaction between P-selectin and PSGL-1 is recognized as a promising therapeutic strategy for thromboinflammatory diseases. A range of P-selectin inhibitors advanced to clinical investigation but were discontinued due to suboptimal efficacy. P-selectin monoclonal blocking antibodies, although approved for sickle cell disease, entail high manufacturing costs and a propensity to elicit anti-drug antibody immune responses, potentially leading to loss of therapeutic efficacy in patients receiving repeated or long-term dosing [11,12]. Improving the binding affinity of existing P-selectin inhibitors or discovering more potent novel inhibitors remains a key challenge.

The native FG derived from sea cucumbers exhibits complex structural characteristics. Following breakthroughs in structural elucidation techniques, a diverse array of oligosaccharides with distinct structural types have been obtained. Our research team has established the FG oligosaccharide compound library, which is dedicated to pharmacological and pharmacodynamic investigations aimed at identifying novel therapeutic targets and mechanisms of action. In the present study, we characterized over ten oligosaccharides from the library and screened a potent P-selectin inhibitor, Ta-9-2 (Mw, 2724), a nonasaccharide featuring a novel disaccharide side chain (Gal_4S6S_-α1,2-L-Fuc_3S_-α1,3). Ta-9-2 inhibited the interaction between P-selectin and sLe^X^ with an IC_50_ of 30.26 μM (95% CI, 22.42–40.86) and a *K*i of 13.86 μM (95% CI, 10.59–18.13). It also suppressed the binding of P-selectin to HL-60 cells with an IC_50_ of 41.24 μM (95% CI, 33.73–50.42) and directly interacted with P-selectin with a *K*_D_ of 83.92 ± 1.084 nM. These inhibitory activities are comparable to or superior to those of the well-characterized P-selectin inhibitors reported to date, including GSnP-6 (IC_50_, 14–28 μM) [26,27,28], Bimosiamose (TBC1269, IC_50_, 70 μM) [29], PSI-697 (IC_50_, 125 μM) [30,31], and Rivipansel (GMI-1070, IC_50_, 423 μM) [32].

It has been reported that the C-type lectin domain of P-selectin serves as the primary carbohydrate-recognition site, and that the core 2 O-glycan bearing sialic acid and fucose on its ligand PSGL-1 are the key motif responsible for P-selectin binding [33,34]. The glycomimetic drug Cylexin (CY-1503) was rationally designed based on this structural motif, but its Phase II clinical trial was discontinued due to suboptimal therapeutic efficacy [11,35]. Subsequent studies revealed that sulfation of three tyrosine residues at positions 46, 48, and 51 of PSGL-1 is critical for affinity enhancement [10,30,31], prompting the design of glycopeptide analogs—rivipansel (3), structurally optimized from rivipansel; after optimization, the IC_50_ value for inhibiting P-selectin-sLe^X^ binding was reduced from 423 μM to 82 μM [13]. The FG oligosaccharide Ta-9-2 exhibits stronger P-selectin inhibitory activity than the optimized rivipansel (3), with a nanomolar-level direct binding affinity to P-selectin. SAR analysis indicated that glycan chain length, the novel disaccharide side chain (Gal_4S6S_-α1,2-L-Fuc_3S_-α1,3), and the sulfation pattern (Fuc_2S4S_) all modulate P-selectin binding. The high affinity of Ta-9-2 for P-selectin (*K*_D_, 83.92 nM) is postulated to arise from the following mechanism: the FG oligosaccharide backbone, together with multiple fucose side chains, adopts a specific spatial conformation under optimal chain length conditions; this conformation may facilitate anionic clustering of sulfate groups around the glycan scaffold, thereby generating a dense electrostatic field that strongly engages the cationic binding pocket of P-selectin.

Additionally, this work still has some limitations. The three assays in vitro used to evaluate the inhibitory activity of the oligosaccharide series against P-selectin reveal a well-defined and consistent structure–activity relationship. Nevertheless, there is the large disconnect between Ta-9-2′s affinity (*K*_D_, 83.92 nM) for P-selectin and its IC_50_ against P-selectin–HL-60 cells (41.24 μM). Some in vivo animal models (for instance, the sickle cell vaso-occlusive model or sepsis-induced thrombosis model) are needed to further evaluate the preventive or therapeutic effects of FG oligosaccharides.

In summary, this work demonstrates the feasibility of screening potent P-selectin inhibitors from naturally depolymerized oligosaccharides and provides a theoretical basis for the structural optimization and novel design of P-selectin inhibitors.

## 4. Materials and Methods

### 4.1. Materials and Reagents

Serious proteins and antibodies purchased from R&D Systems (Minneapolis, MN, USA) are as follows: Recombinant human P-selectin Fc chimeras (P-selectin, 137-PS); Recombinant Human IgG1 Fc (hIgG, 110-HG); Recombinant Human PSGL-1 Fc Chimera (PSGL-1, 3345-PS); P-selectin-blocking monoclonal antibody (Anti-hp, BBA30); and Human IgG Fc PE-conjugated Antibody (PE-anti-hIgG, FAB110P). Bovine albumin (BSA, 7906-25) was sourced from BioVision Technology (Milpitas, CA, USA). HRP-conjugated goat anti-human IgG (SA00001-17) was sourced from Proteintech (Wuhan, China). RPMI 1640 Medium (61870036), Fetal Bovine Serum (FBS, 10099-141), and penicillin-streptomycin (10378016) were sourced from Gibco, Thermo Fisher Scientific (Waltham, MA, USA).

3,3′,5,5′-Tetramethylbenzidine substrate solution (TMB, P0209) was sourced from Beyotime Biotechnology (Shanghai, China). PBS Tablets (092810305) were purchased from MP Biomedicals (Solon, OH, USA). Sialyl Lewis X tetrasaccharide (sLe^X^, S1782-1MG) was sourced from Sigma-Aldrich (Shanghai, China). Sialyl Lewis X-polyacrylamide-biotin (sLe^X^-PAA-B, 01-045) was sourced from GlycoTech Corporation (Gaithersburg, MD, USA). Low molecular weight heparin (LMWH, Enoxaparin, Mw 4500 Da, Sanofi-Aventis, Chilly-Mazarin, France) and Fondaparinux sodium (Fpx, Mw 1728 Da, GlaxoSmithKline, Brentford, UK) were purchased from Sinopharm (Beijing, China). All other chemical reagents were sourced from Sigma-Aldrich.

Pierce™ Streptavidin-coated high-capacity plates (15501) were sourced from Thermo Fisher Scientific (Waltham, MA, USA). Black solid 96-well flat bottom plates (655209) were sourced from Greiner bio-one (Frickenhausen, Germany). Protein A sensors (18-5010) were sourced from Sartorius (Shanghai, China).

### 4.2. Preparation of Oligosaccharides

A native FG was isolated and purified from the sea cucumber *H. fuscopunctata*. Low-molecular-weight fragments were generated from native FG via β-eliminative depolymerization, followed by preparative gel filtration and anion-exchange chromatography. Well-defined oligosaccharides (HS-17, HS-14, HS-11, HS-8, HS-5, and HS-3A) were subsequently isolated and structurally characterized from the depolymerized products, as reported previously [18,19].

The oligosaccharide preparation protocol for sea cucumber *Thelenota ananas* closely parallels that established for *H. fuscopunctata*, with the key modification being the incorporation of a controlled mild acid hydrolysis step. This additional treatment enables the selective cleavage of labile fucosyl branches, thereby facilitating detailed structural elucidation (particularly of heterogeneous or highly substituted side chains), as documented in prior studies [22].

### 4.3. Microplate Binding Assay

Streptavidin-coated high-capacity plates were washed by adding 300 μL washing buffer (PBS supplemented with 1 mM CaCl_2_, 1 mM MgCl_2_, and 0.1% Tween-20). A total of 100 μL of 1 μg/mL sLe^X^-PAA-B or sample dilution buffer (PBS containing 1 mM CaCl_2_, 1 mM MgCl_2_, and 1% BSA) was added and incubated at 37 °C for 1 h. Plates were then washed five times.

Subsequently, in saturation binding experiments, a concentration series of recombinant P-selectin or hIgG was added (100 μL/well) and incubated at 37 °C for 1 h, followed by five washes; HRP-anti-hIgG (1:2000 dilution) was then added and incubated at 37 °C for 30 min. The plate was washed, and next, 100 μL of TMB substrate solution was added. After 10 min at room temperature in the dark, the reaction was terminated with 100 μL of 2 M H_2_SO_4_. Absorbance at 450 nm was immediately measured using a BioTek ELX808 microplate reader (BioTek, Winooski, VT, USA). In competitive experiments, a single concentration of P-selectin (1 μg/mL), or a mixture of P-selectin and serial concentrations of inhibitors, was added to each well; all subsequent steps were identical.

The binding rate was calculated by normalizing each value to the P-selectin group (without competitors). The IC_50_ of the inhibitor was determined by fitting the log (inhibitor) vs. response curves equation (a 4-parameter dose response curve). The *K*_D_ of P-selectin-sLe^X^ was determined by fitting the binding-saturation curve using one site-fit total and nonspecific binding equation, which is essential for calculating the *K*_i_, and the *K*_i_ of the inhibitors were determined by fitting the one site-fit *K*_i_ equation. The statistical models described above were created using GraphPad Prism 10.0 software.

### 4.4. Flow Cytometry Binding Assay

HL-60 cells were obtained from the Kunming Cell Bank, Chinese Academy of Sciences (KCB2014051YJ). HL-60 cells were maintained in RPMI 1640 medium supplemented with 10% fetal bovine serum (FBS) and 1% penicillin-streptomycin mixture (100 U/mL penicillin, 100 μg/mL streptomycin) in a humidified CO_2_ incubator at 37 °C with 5% CO_2_, and the culture medium was refreshed every 2 days.

Fifty microliters of HL-60 cells (5 × 10^5^ cells per sample) were mixed with P-selectin (final concentration: 1.25 μg/mL) and serial concentrations of inhibitors (FG oligosaccharides, sLe^X^, LMWH, Anti-hp, PSGL-1) or vehicle (a working buffer composed of PBS supplemented with 1 mM CaCl_2_, 1 mM MgCl_2_, and 0.5% BSA); the total volume was adjusted to 100 μL with working buffer. Negative control groups were established by treating 50 μL of HL-60 cells with vehicle alone or with hIgG to account for non-specific binding. Following incubation at 37 °C for 30 min, samples were added to 500 μL of working buffer and centrifuged at 300× *g*; the wash step was repeated twice. Subsequently, 5 μL of PE-anti-hIgG was added to a sample tube, for a total volume of 100 μL with working buffer. After incubating at room temperature (25 °C) for 30 min in the dark, the cells were washed twice again. Cells were resuspended in 300 μL of working buffer and immediately analyzed using a FACSCelesta flow cytometer (BD Biosciences, Milpitas, CA, USA). A total of 50,000 events were acquired per sample; the mean fluorescence intensity (MFI) was calculated using FlowJo 10.9.0 software. The data were calculated after subtracting the value of the negative control (hIgG), and results were expressed as the percentage of control (the sample treated with P-selectin without inhibitor). The IC_50_ of the inhibitor was determined by fitting the log (inhibitor) vs. response curves equation (a 4-parameter dose response curve) using GraphPad Prism 10.0 software.

### 4.5. Biolayer Interferometry-Based Binding Assays

All experiments were conducted using an Octet Red 96 instrument (Sartorius, Bohemia, NY, USA). Assays were monitored in real-time at 30 °C in black, solid 96-well flat bottom plates with agitation set to 1000 r/min. P-selectin protein was loaded onto the Protein A sensors until saturation in PBST (0.02% Tween 20 in PBS). Sensors loaded with P-selectin were equilibrated for 300 s in running buffer (PBS containing 1 mM CaCl_2_, 1 mM MgCl_2_, 0.02% Tween 20, and 0.1% BSA, pH 7.35) and then associated with gradient concentrations of FG oligosaccharides or buffer for 300 or 900 s, followed by a dissociation phase of 300 s in running buffer. Response values are reported after subtracting the value of the blank control. Biolayer interferometry kinetic data were analyzed using the Octet Analysis Studio 13 software and the equilibrium dissociation constant (*K*_D_) was determined by fitting the binding curves globally using a 1:1 binding model.

### 4.6. Statistical Analysis

Statistical analysis was performed using GraphPad Prism 10. All data in this article are expressed as the mean ± 95% confidence interval (CI), and the SE is used when the 95% CI cannot be calculated. Significance of IC_50_ differences from dose-inhibition curves is assessed by 95% confidence interval (CI) comparison: non-overlapping CIs indicate statistical significance; for partially overlapping CIs, Log IC_50_ values are compared using nonlinear regression (*p* < 0.05 considered significant).

## Figures and Tables

**Table 1:** The IC_50_ of FG oligosaccharides for inhibiting the binding of P-selectin to sLe^X^ or HL-60 cells.

Samples	Branch Types	Mw (Da)	P-Selectin/sLe^X^		P-Selectin/HL-60 Cells
			IC_50_ (95% CI) ^a^ (μM)	*K*_i_ (95% CI) ^b^ (μM)	IC_50_ (95% CI) ^a^ (μM)
HS-3A	Fuc_3S4S_	958	>1000	779.3 (351.2–1729)	>1000
HS-5	Fuc_3S4S_	1506	701.1 (440.7–1116)	498.5 (277.4–894.3)	>1000
HS-8	Fuc_3S4S_	2462	179.1 (149.2–215.1)	88.47 (72.10–108.5)	>1000
HS-11	Fuc_3S4S_	3417	66.17 (40.29–108.7)	31.60 (19.78–50.51)	260.5 (90.50–749.6)
HS-14	Fuc_3S4S_	4373	23.55 (18.98–29.22)	11.10 (8.625–14.29)	78.77 (38.92–113.6)
HS-17	Fuc_3S4S_	5328	16.62 (12.56–21.98)	7.582 (5.548–10.36)	71.03 (41.22–122.4)
Ta-3-1	Fuc_2S4S_	958	514.8 (96.68–2741)	358.4 (157.9–813.2)	/ ^d^
Ta-4	Gal_4S6S_-α1,2-L-Fuc_3S_-α1,3	1222	>1000	>1000	/ ^d^
Ta-5	Fuc_2S4S_	1506	249.2 (84.11–738.3)	105.5 (62.67–177.4)	>1000
Ta-6-2	Gal_4S6S_-α1,2-L-Fuc_3S_-α1,3	1770	128.1 (80.87–202.8)	69.07 (43.99–108.5)	>1000
Ta-6-1	Fuc_2S4S_	1913	225.3 (117.1–600.7)	141.3 (71.66–278.6)	>1000
Ta-7	Gal_4S6S_-α1,2-L-Fuc_3S_-α1,3	2177	76.98 (37.00–160.2)	29.95 (19.24–46.62)	109.2 (54.45–290.3)
Ta-8	Fuc_2S4S_	2462	125.5 (101.4–155.5)	73.38 (52.36–102.9)	97.54 (51.42–216.4)
Ta-9-2	Gal_4S6S_-α1,2-L-Fuc_3S_-α1,3	2726	30.26 (22.42–40.86)	13.86 (10.59–18.13)	41.24 (33.73–50.42)
Ta-9-1	Fuc_2S4S_	2869	89.02 (58.88–134.6)	40.46 (29.47–55.54)	251.1 (116.2–542.7)
sLe^X c^		821	>1000	>1000	32.64% at 1.2 mM
Fpx ^c^		1728	247.6 (79.60–770.3)	182.0 (54.54–607.6)	/ ^d^
LMWH ^c^		~4500	40.23 (21.41–152.8)	17.68 (12.04–37.62)	90.20 (53.10–165.9)
P-selectin blocking antibody ^c^		150 kD	3.841 nM (3.105–4.752)	1.314 nM (0.3249–4.453)	4.421 nM (4.103–4.762)
PSGL-1 ^c^		105.2 kD	262.2 nM (106.7–609.5)	133.9 nM (22.98–554.5)	20.49 nM (18.17–23.10)

^a^ IC_50_, Half-maximal inhibitory concentration. IC_50_ presented as mean and 95% confidence interval (CI). ^b^ *K*_i_, inhibition dissociation constant. *K*_i_ presented as mean and 95% CI. ^c^ Positive control, for cross-referencing with P-selectin inhibitors developed by other independent research teams. ^d^ Not detected.

## Data Availability

All data needed to evaluate the conclusions in this article are included in this main text. Additional data related to this article are available from the corresponding author upon reasonable request.

## Abbreviations

The following abbreviations are used in this manuscript:

Anti-hp	P-selectin-blocking monoclonal antibody
BLI	Biolayer interferometry
CI	confidence interval
FCS	Fucosylated chondroitin sulfate
FG	Fucosylated glycosaminoglycan
Fpx	Fondaparinux sodium
FucS	sulfated α-L-fucose
Gal	galactose
GalNAc	Acetyl galactosamine
GlcA	Glucuronic acid
hIgG	Recombinant Human IgG1 Fc
IC_50_	half-maximal inhibitory concentration
*K*_D_	Equilibrium dissociation constant
*K*_i_	Inhibition dissociation constant
*k*_on_	Association rate constant
*k*_off_	Dissociation rate constant
LMWH	Low molecular weight heparin
MFI	Mean fluorescence intensity
Mw	Molecular weight
PSGL-1	P-selectin glycol-protein ligand-1
SAR	Structure-activity relationship
sLe^X^	Sialyl Lewis X
sLe^X^-PAA-B	Sialyl Lewis X-polyacrylamide-biotin
